# Management and outcome of intracardiac heartworms in dogs

**DOI:** 10.1186/s13071-023-05677-5

**Published:** 2023-04-28

**Authors:** Jorge Vila, Elisabeth Alost

**Affiliations:** Medvet New Orleans, Metairie, LA USA

**Keywords:** Heartworm disease, Intracardiac heartworms, Caval syndrome, Dogs, Pimobendan, Sildenafil

## Abstract

**Background:**

Intracardiac heartworm (IH) disease is a serious condition that can become life threatening if the patient develops caval syndrome. We aim to describe the management and outcome of IH in dogs evaluated by Medvet’s New Orleans cardiology service from November 2015 to December 2021.

**Methods:**

Records of 27 dogs with IH were examined retrospectively. Follow-up information was obtained from phone conversations with referring veterinarians and owners.

**Results:**

Nine of 27 dogs had a previous diagnosis of heartworm disease and were undergoing “slow kill” treatment; 12/27 dogs' heartworm disease was a new diagnosis, and 6/27 had either scheduled or started adulticide therapy. Nine dogs had heartworm extraction. No dogs died during the heartworm extraction procedure. Four of 9 dogs have died (survival time 1; 676; 1815 and 2184 days). One dog died the day after the procedure secondary to continued respiratory distress; the other three died of non-cardiac causes. Five of nine are alive (median follow-up 1062 (range 648–1831) days. Eleven dogs had IH resolution. In 7/11 this occurred while undergoing stabilization for heartworm extraction. In 4/11 heartworm extraction was not recommended because of low IH burden. All dogs with IH resolution were discharged from the hospital. Four of 11 have died (survival time 6; 22, 58 and 835 days), and 6/11 are alive (median follow-up 523 (range 268–2081) days. One was lost to follow-up after 18 days. Five dogs were medically managed. In one of five dogs, extraction was not recommended because of low IH burden. In four of five extraction was recommended but declined. One of five has died (survival 26 days), and four of five are alive (follow-up 155, 371, 935 and 947 days). Two dogs were killed at the time of diagnosis. Fifteen of 27 dogs were considered to have caval syndrome.

**Conclusion:**

The results suggest that patients with IH resolution have a good long-term prognosis. Most often IH resolution occurred while the dog was undergoing stabilization for heartworm extraction. When IHs are present, heartworm extraction should still be considered the treatment of choice and recommended as first-line therapy whenever possible.

**Graphical Abstract:**

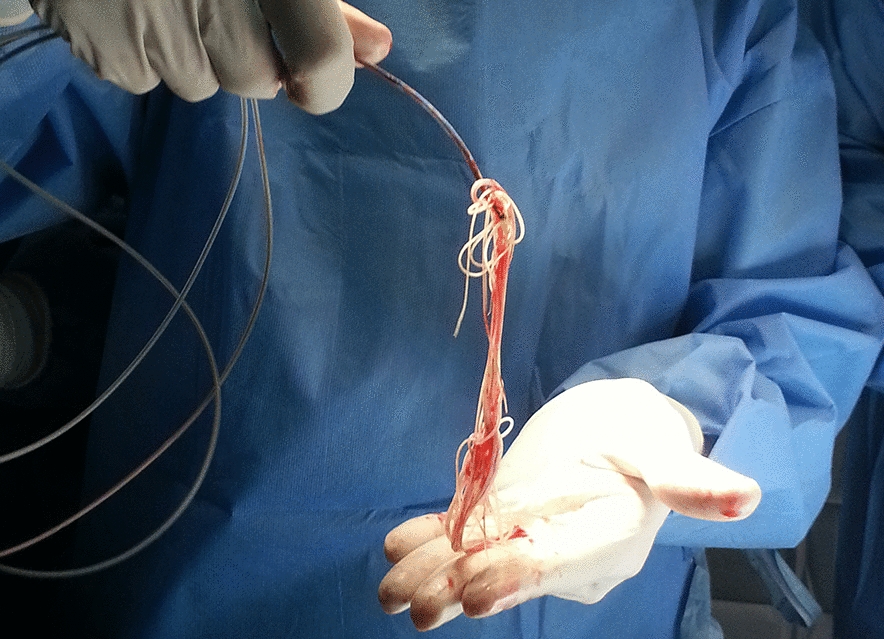

## Introduction

Intracardiac heartworm (IH) disease is a serious condition that can become life threatening if the patient develops caval syndrome. It is the result of retrograde movement of the worms from the pulmonary arteries into the right cardiac chambers and potentially even the vena cava [1–5]. The presence of heartworms in the right heart chambers can then cause obstruction of blood flow and result in red blood cell lysis. While not all dogs with intracardiac heartworms develop caval syndrome, the presence of anemia and/or hemoglobinuria in a patient with intracardiac heartworms is consistent with caval syndrome [5]. Heartworm extraction results in resolution of the hemoglobinuria and anemia. Extraction is often performed under fluoroscopic and/or echocardiographic guidance by inserting a retrieval basket or other retrieval device into the right heart chambers via the jugular vein [1]. The reported outcome for extraction varies between 50 and 67% success [2, 4]. Often these patients are hemodynamically unstable, and it has been suggested that taking measures to help stabilize them prior to extraction may improve outcomes [1, 6].

Resolution of intracardiac heartworms has previously been reported [1, 4, 7]. Treatment with sildenafil, pimobendan and oxygen therapy was believed to improve hemodynamics and right heart function potentially aiding in the movement of the heartworms back into the pulmonary artery [1, 7]. However, these reports did not provide causality, and intracardiac heartworm resolution due to other reasons cannot be excluded [1, 7]. We aim to describe the management and outcome of intracardiac heartworms in dogs evaluated by Medvet’s New Orleans cardiology service from November 2015 to December 2021.

## Methods

Records of 27 dogs with IH were examined retrospectively. For each patient, signalment historical information, clinical signs at presentation, month of presentation, physical examination findings, medical therapy, clinicopathology and results of the echocardiographic examination were evaluated. All patients had an echocardiogram performed with a Phillips Cx50 ultrasound machine by a single board-certified cardiologist confirming the presence and/or resolution of intracardiac heartworms. Intracardiac heartworm burden was subjectively estimated by the cardiologist. The patient was considered to have low worm burden if only a small number of worms was estimated (< 5 worms) or high worm burden if a larger number of worms was noted filling the right ventricle or right atrium. Follow-up information was obtained from phone conversations with referring veterinarians and owners. Anemia was defined as a pack cell volume (PCV) < 37% and thrombocytopenia as < 175,000 platelets. Cases were considered to be in caval syndrome if they had anemia and/or pigmenturia along with clinical signs consistent with caval syndrome such as anorexia, dyspnea, weakness, weak pulses and/or jugular vein distention. Dogs were divided into categories based on treatment as follows: heartworm extraction (patients that underwent transvenous heartworm extraction), intracardiac heartworm resolution (patients in which the heartworms moved back into the pulmonary artery), medical management (patients that still had intracardiac heartworms and underwent heartworm adulticide therapy) or euthanasia at the time of presentation. Heartworm extraction was performed under general anesthesia by or under the guidance of a board-certified anesthesiologist. Anesthetic protocols were tailored to each patient. Most often patients received premedication with hydromorphone/midazolam or methadone/midazolam followed by propofol induction and maintenance with either isoflurane or sevoflurane. All extractions were performed by a single board-certified cardiologist under fluoroscopic guidance inserting a retrieval basket (Olympus Flower basket FG-301Q) into the right heart chambers via the right jugular vein. During worm extraction, echocardiogram was performed periodically to ensure that the worms were removed from the right heart chambers. Descriptive statistics, mean, median, standard deviation and range, were calculated. Data were compared between the heartworm extraction and the intracardiac heartworm resolution patients. Normality for continuous variables was assessed using the Shapiro-Wilk test. Normally distributed data are reported as mean ± SD and not normally distributed data as median and range. Continuous data were compared using a Student’s t test if normally distributed or Mann-Whitney U test if not normally distributed. Categorical data were compared with Fisher’s exact test. Survival analysis was performed with the log-rank test. Statistical significance was considered as *P* < 0.05. Only descriptive statistical analysis was used for the medical management and euthanasia groups. All statistical analysis was performed with GraphPad Prism, a commercially available software package.

## Results

Twenty-seven dogs were identified to have been evaluated by the cardiology service and have intracardiac heartworm disease. Nine dogs had heartworm extraction, 11 had intracardiac heartworm resolution, 5 were medically managed, and 2 were euthanized at the time of presentation. The median age was 4.5 (range 1–11) years. There was no statistically significant difference in age between the extraction and IH resolution group (Mann-Whitney, *P* = 0.33). The median weight was 8.6 (range 1.57–29.7) kg. There was no statistically significant difference in weight between the extraction and IH resolution group (Mann-Whitney, *P* = 0.82). The breeds of dogs included Chihuahua (*n* = 8), terrier mix (*n* = 2), Miniature Pinscher (*n* = 2), Mixed (*n* = 2) and one each of English Bulldog, American Pit Bull terrier, Brittany Spaniel, Boxer mix, Husky mix, Dachshund, Australian Shepherd, Chow Chow mix, Boston Terrier, Labrador Retriever, Pug, Yorkshire Terrier and Hound. The frequency of case presentation was divided into seasons with three cases presenting in the spring (March, April, May), 10 cases in the summer (June, July and August), four cases in the fall (September, October and November) and 10 cases in the winter (December, January and February).

At the time of presentation, nine dogs had a previous diagnosis of heartworm disease and were undergoing “slow kill” treatment, i.e. treatment with a macrocyclic lactone with or without doxycycline instead of adulticide therapy; for 12 dogs heartworm disease was a new diagnosis, and six dogs had started doxycycline, a macrocyclic lactone and scheduled treatment (*n* = 4) or received one single dose of melarsomine (*n* = 2). For the two patients that received melarsomine, one followed the American Heartworm Society guidelines. In the other patient, treatment was recommended per the guidelines but for financial reasons the owner elected to administer doxycycline and monthly oral ivermectin/pyrantel. After approximately 18 months, they pursued adulticide therapy. Right-sided congestive heart failure and intracardiac heartworms were diagnosed approximately 2 weeks after the melarsomine injection. Time of “slow kill” treatment was available for seven dogs with a median treatment time of 8 (range 2–24) months. Information on the specific macrocyclic lactone during treatment was available for three cases. All were receiving a licensed heartworm preventative at the label dose. One case had received topical imidacloprid plus moxidectin for 8 months, and the other two had received oral ivermectin/pyrantel for 5 and 24 months. Fifteen dogs (55.5%) were considered to be in caval syndrome: four of the nine (44.4%) that were undergoing slow kill treatment, nine of the 12 (75%) that were newly diagnosed with heartworm disease, and two of the six (33.3%) that were undergoing heartworm treatment. The clinical characteristics are reported by group in Table 1. The clinical signs at presentation were as follows: lethargy in eight of 27 (29.6%), pigmenturia in five of 27 (18.5%), cough in seven of 27 (25.9%), dyspnea in 12 of 27 (44.4%), ascites in 10 of 27 (37%) and syncope in five of 27 (18.5%). Intracardiac heartworm resolution patients presented with dyspnea more often than patients that had heartworm extraction (Fisher’s exact test *P* = 0.01, OR = 21.3, 95% CI = 2.15–259). Anemia was noted in 14 of 21 patients (66.7%). There was no difference in the frequency of anemia between the groups. However, heartworm extraction patients had a lower PCV 24.1% (SD ± 11.15%, range 12.4–40%) compared to the intracardiac heartworm resolution patients with a PCV of 38.23% [SD ± 9.44%, range 25–60%; *T*-test, *t*(16) = 2.909, *P* = 0.01]. Ten of 20 patients (50%) had thrombocytopenia. Intracardiac heartworm burden was considered high in 18 of 27 (66.6%) patients.

**Table 1:** Clinical characteristics

Characteristic	All dogs	Extraction	Resolution	Medical	Euthanasia	Statistical analysis
Number	27	9/27	11/27	5/27	2/27	N/A
Lethargy	8/27	2/9	2/11	3/5	1/2	Fisher’s exact test *P* ≥ 0.99
Pigmenturia	5/27	1/9	2/11	2/5	0/2	Fisher’s exact test *P* ≥ 0.99
Dyspnea	12/27	1/9	8/11	2/5	1/2	Fisher’s exact test *P* = 0.01 OR = 21.3, 95% CI = 2.15–259
Ascites	10/27	4/9	3/11	2/5	1/2	Fisher’s exact test *P* = 0.64
Syncope	5/27	1/9	2/11	2/5	0/2	Fisher’s exact test *P* ≥ 0.99
Anemia (PCV < 37%)	13/21	7/9	5/9	0/1	1/2	Fisher’s exact test *P* = 0.62
Thrombocytopenia (< 175,000)	10/20	4/9	4/8	0/1	2/2	Fisher’s exact test *P* ≥ 0.99
High worm burden	18/27	8/9	6/11	2/5	2/2	Fisher’s exact test *P* = 0.16
Caval syndrome	15/27	7/9	4/11	3/5	1/2	Fisher’s exact test *P* = 0.09

Statistical analysis refers to comparison between extraction and resolution group

*N/A* not applicable

Nine dogs had heartworm extraction. Six dogs had surgery 1 day after presentation, with two dogs having surgery the same day and one dog having surgery 2 days after presentation. All nine patients received sildenafil, and eight received pimobendan prior to heartworm extraction. The median dose of sildenafil was 2.2 (range 0.8–3.2) mg/kg Q8 h PO. Two patients received sildenafil twice a day. The median pimobendan dose was 0.25 mg/kg Q12 h PO (range 0.16 mg/kg Q8 h PO to 0.58 mg/kg Q12 h PO). Two patients received pimobendan three times a day. Other treatment administered prior to extraction included: clopidogrel (*n* = 1), oxygen (*n* = 3), intravenous fluids (*n* = 2), doxycycline (*n* = 4), fresh frozen plasma (*n* = 1) and blood transfusion (*n* = 6). One dog received vitamin K in the hospital prior to the diagnosis of caval syndrome. All patients received dexamethasone sodium phosphate 0.2 mg/kg IV and diphenhydramine 2 mg/kg IM prior to extraction. Extraction time was available for six patients with a median time of 60 (range 55–120) min. No dogs died during the heartworm extraction procedure. Median heartworm number extracted was 27 (range 1–63). Five dogs were discharged 1 day after heartworm extraction, and 3 dogs were discharged 2 days after extraction. The median hospitalization time was 2 (range 1–3) days. Eight of nine patients were discharged from the hospital. Medication recommended at discharge included: pimobendan (*n* = 8), sildenafil (*n* = 8), prednisone (*n* = 4), doxycycline (*n* = 7), furosemide (*n* = 4) and tramadol (*n* = 3). Four of nine have died (survival time 1, 676, 1815 and 2184 days). One dog died the day after the procedure secondary to continued respiratory distress; the other three died of non-cardiac causes (pneumonia *n* = 1; lymphoma *n* = 2). Five of nine are alive [median follow-up time 1062 (range 648–1831) days].

Eleven dogs had IH resolution. Eight patients presented with dyspnea (three with suspected pulmonary thromboembolism, three with pneumonitis and one patient suspected to have both). In one patient the reason for dyspnea was not listed in the record, and referral radiographs were not available for review. In seven of 11 (63.6%) IH resolution occurred while undergoing stabilization for heartworm extraction. The time to IH resolution was 1 day in five of these seven patients. In the other two patients, resolution was noted approximately 2 h after administration of sildenafil in one patient and 5 days later in the other when he returned for surgery. In four of 11 heartworm extraction was not recommended because of low IH burden. Three of these patients were hospitalized for dyspnea (two for suspected pulmonary thromboembolism and one with pneumonitis), and intracardiac heartworm resolution was noted at days 3, 4 and 6 when the cardiology service was available to perform a repeat evaluation. In the fourth case resolution was noted 13 days later when the patient returned for follow-up of right-sided heart failure due to pulmonary hypertension. All 11 patients received sildenafil and nine of 11 patients received pimobendan prior to IH resolution. The median dose of sildenafil was 2.2 (range 1–3.2) mg/kg Q8 h PO. Two patients received sildenafil twice a day. The median pimobendan dose was 0.34 (range 0.22–0.67) mg/kg Q12 h PO. Other treatment performed in the hospital included: clopidogrel (*n* = 2), oxygen (*n* = 8), intravenous fluids (*n* = 2), doxycycline (*n* = 6), corticosteroids (*n* = 8), enrofloxacin (*n* = 1), furosemide (*n* = 1), abdominocentesis (*n* = 1) and vitamin k (*n* = 1). All dogs with IH resolution were discharged from the hospital. Medication recommended at discharge included pimobendan (*n* = 9); sildenafil (*n* = 11), prednisone (*n* = 9), doxycycline (*n* = 9), furosemide (n = 3) and clopidogrel (*n* = 1). There was no significant difference in the frequency of IH resolution between dogs that had high versus low intracardiac heartworm burden (Fisher’s exact test *P* = 0.43). Four of 11 have died (two were euthanized because of poor quality of life associated with dyspnea at day 6 and day 22; one died from a suspected pulmonary thromboembolism at day 58 1 month after the first melarsomine injection and one died from pulmonary hypertension and right-sided congestive heart failure at day 835). Six of 11 are alive [median follow-up 523 (range 268–2081) days]. One was lost to follow-up after 18 days. There was no difference in the probability of survival between the heartworm extraction and heartworm resolution groups (heartworm extraction: 2184 days versus resolution 835 days, log-rank test, *x*^2^ = 0.991, df = 1, *P* value = 0.32).

Five dogs were medically managed. In one dog, extraction was not recommended because of low IH burden. In four of five extraction was recommended but declined. All patients received sildenafil, and three of five received pimobendan. The median dose of sildenafil was 1.85 (range 1.1–2.2) mg/kg Q8 h PO. One patient received sildenafil twice a day. For the three patients that received pimobendan, the dose was 0.26 mg/kg Q12 h PO, 0.27 mg/kg Q12 h PO and 0.31 mg/kg Q12 h PO. Other treatment performed in the hospital included thoracocentesis (*n* = 2), corticosteroids (*n* = 3), doxycycline (*n* = 2) and furosemide (*n* = 2). All patients were discharged from the hospital. Medication recommended at discharge included pimobendan (n = 3), sildenafil (*n* = 5), prednisone (*n* = 2), doxycycline (*n* = 2) and furosemide (*n* = 2). It was recommended that treatment with melarsomine be performed as soon as possible with the referring veterinarian. One patient died (survival of 26 days) without following up for treatment with melarsomine. Four of five are alive (follow-up time of 155, 371, 935 and 947 days). The referring veterinarians confirmed completion of heartworm treatment in three of these patients. The fourth patient started doxycycline from his primary care veterinarian but never returned for treatment.

Two dogs were euthanized at the time of diagnosis. One patient presented with dyspnea and abdominal effusion. This patient had a known history of heartworm disease and had been receiving oral ivermectin/pyrantel for 24 months. The other patient presented for weakness and was noted to be anemic and thrombocytopenic and had an elevated total bilirubin. This patient had a new diagnosis of heartworm disease and was in caval syndrome. Both patients had high worm burden, and heartworm extraction was recommended. Euthanasia was elected in both because of inability to pursue extraction due to finances and perceived poor prognosis without extraction. As mentioned previously, 15 dogs were considered to be in caval syndrome. Heartworm extraction was recommended for all 15 cases. Seven had heartworm extraction, four had intracardiac heartworm resolution prior to extraction, three were medically managed and one was euthanized. Patients with a high intracardiac worm burden were more likely to have caval syndrome compared to those with low intracardiac worm burden (Fisher’s exact test *P* = 0.037 OR = 9.1, 95% CI = 1.38–49.86).

## Discussion

We aim to describe the management and outcome of intracardiac heartworms in dogs. The study described the management of 27 intracardiac heartworm disease cases. While a wide range of breeds presented with intracardiac heartworms, Chihuahuas were the most common breed seen (29.6%). An increased predisposition for small breed dogs developing IH disease has previously been reported [5]. In this study Chihuahuas were also overrepresented in a population of patients with IH disease [5]. It was suggested that the relatively small size of these dogs in relation to the length of the worms may predispose them to worm migration into the right heart chambers [5]. Most commonly the patients presenting with intracardiac heartworm had a new diagnosis of heartworm disease. Heartworm disease is enzootic in North America with a particularly higher prevalence in the southern USA, especially along the Gulf Coast and Mississippi Delta [3, 8, 9]. A study evaluating the prevalence of canine heartworm infection in Mississippi animal shelters showed that 34.4% of the serum samples evaluated were heartworm antigen positive [10]. The fact that most of the cases in our series were either undiagnosed or a very recent diagnosis emphasizes the importance of yearly heartworm testing and heartworm prevention. Heartworm prevention, early diagnosis and treatment could help avert development of IH disease and its potentially serious and life-threatening complications.

We observed a bimodal presentation of dogs with IH disease with most cases being seen in the winter or summer. This is an unforeseen finding given the warm climate and the yearlong prevalence of heartworm disease in the Gulf Coast area. A previous retrospective study showed no difference in heartworm extraction frequency in a similar geographic location [5].

In our population, 15 of the 27 patients evaluated were considered to have caval syndrome. It is important to highlight the difference between caval syndrome versus intracardiac heartworms. Intracardiac heartworm disease occurs from retrograde displacement of the worms from the pulmonary arteries into the right heart [1, 11]. In some patients, this leads to obstruction of blood flow by the worms resulting in damage and lysis of the red blood cells; this subsequently causes the anemia, pigmenturia and clinical signs associated with caval syndrome [2, 11]. However, not all patients with intracardiac heartworm disease appear to develop these signs [5, 7]. It is not completely understood why some patients go on to develop caval syndrome while others have intracardiac heartworms without the typical caval syndrome signs. Heartworm burden and decreased cardiac output as a result of pulmonary hypertension and right heart dysfunction likely contribute to the development of caval syndrome [1, 2, 4, 11]. Intracardiac heartworm burden is likely an important determining factor for the development of caval syndrome. In a previous study, patients with estimated high intracardiac worm burden were more likely to have anemia, pigmenturia and bilirubinuria [5]. While not all patients with high intracardiac heartworm burden develop caval syndrome, in our population, these patients were more likely to be in caval syndrome. Some of the cases with high intracardiac heartworm burden without caval syndrome may represent a spectrum of progression with more signs associated with caval syndrome developing with time as the intracardiac heartworms remain in the right heart chambers.

At our institution, heartworm extraction is recommended in all patients considered to have a high heartworm burden regardless of the presence or absence of caval syndrome signs. Extraction is often not performed immediately but after a period of stabilization. In our population this was most often the next day after presentation. This time was likely affected by multiple factors including patient’s clinical signs, hemodynamic status as well as availability of the cardiology service to perform the procedure. In preparation for extraction, patients routinely received sildenafil and pimobendan preoperatively. Successful heartworm extraction outcomes vary between 50 and 67% [2, 4]. Of the nine patients in this study that had extraction, all survived the procedure, and one patient died the day after the surgery because of progressive dyspnea that was present preoperatively. The potential procedural success improvement may be related to availability of pimobendan and sildenafil. The previous procedural success data are likely from a time where these medications were not as widely available. In one report most of the patients did not receive pimobendan or sildenafil preoperatively [4].

Eleven patients had heartworms move back into the pulmonary arteries. Four patients had a low worm burden and surgery was not considered necessary, while in seven of the 11 patients the worms moved while being stabilized for heartworm extraction. Resolution of intracardiac heartworm disease has been reported previously [1, 5, 7]. It has been suggested that treatment with sildenafil, pimobendan and in some cases oxygen therapy may improve hemodynamics and right heart function potentially aiding the movement of the heartworms back into the pulmonary artery [1, 7]. In our population, intracardiac heartworm burden does not appear to be a significant determinant factor in the resolution of intracardiac heartworm disease with a similar number of high worm burden and low worm burden patients having IH resolution. Patients with IH resolution had higher frequency of dyspnea with four out of these eight patients having suspected pulmonary thromboembolism. We presume that this may have been a factor in the development of IH and subsequent IH resolution in these patients. It has long been suspected that retrograde migration of worms can occur secondary to an acute worsening of pulmonary hypertension and reduction of cardiac output [2, 12]. An acute event such as a pulmonary thromboembolism may have contributed to the retrograde migration of worms and the subsequent diagnosis of IH. It is reasonable to assume that treatment with pimobendan, sildenafil and oxygen leads to an improvement of the cardiac output and hemodynamic status in these patients aiding in the migration of the heartworms back into the pulmonary arteries as previously reported [1, 7].

To our knowledge, no data are available on the long-term outcome of patients with IH resolution. Six of 11 patients with IH resolution are known to be alive at the time of this writing with a median follow-up of 523 (range 268–2081) days. Four patients died, and one was lost to follow-up at 18 days. Two of these patients had dyspnea at the time of presentation and were euthanized because of poor quality of life associated with recurrent or exertional dyspnea. One died after long-term treatment for right-sided congestive heart failure and pulmonary hypertension. The fourth patient jumped out of bed and then developed dyspnea, coughed blood and died suddenly approximately 1 month after his first melarsomine injection. This acute event underscores the importance of strict cage rest in patients undergoing heartworm treatment, especially those with advanced heartworm disease [9]. Overall, the patients with intracardiac heartworm resolution had a good long-term prognosis. Notably, in most of these cases surgery was recommended, and the IH resolution occurred while undergoing preparation for heartworm extraction. Resolution usually occurred the day after presentation. However, heartworm extraction was not delayed in an attempt to get the worms back into the pulmonary artery but was scheduled as soon as was feasible once the patient was considered stable.

In five patients, heartworm treatment with melarsomine was recommended despite the patients still having intracardiac heartworms. In four of these patients heartworm extraction was recommended but was not possible for financial reasons. One patient died at 26 days without following up for heartworm treatment. The other four patients survived and are doing well at the time of this writing. Notably, these patients were stable enough where euthanasia was not considered absolutely necessary, and a last ditch attempt to help the patients was made by recommending treatment with sildenafil and sometimes pimobendan followed by heartworm treatment as soon as possible. While four of the patients did well long term, treatment with melarsomine should not be considered as a replacement to heartworm extraction. Most cases with caval syndrome signs would be expected to progress and potentially die from their disease faster than the time it would take for heartworm treatment with melarsomine to be completed and/or take effect [6, 8, 9]. These cases represent a few patients that were stable enough to attempt melarsomine treatment as the last alternative to euthanasia.

The study has several limitations. The retrospective nature of the study limited some of the available information. There is a potential selection bias in the data presented. These cases represent patients seen by the cardiology service and therefore are a fraction of the intracardiac heartworm disease cases that presented to our hospital. Most intracardiac heartworm disease cases present to the emergency department. Most of these are either euthanized or treated medically without pursuing evaluation with the cardiology service. While an exact number cannot be provided, the author would estimate that approximately one out five intracardiac heartworm disease patients seen at our hospital pursues evaluation with the cardiology service.

## Conclusions

Intracardiac heartworm resolution has been previously reported. However, the long-term prognosis of these patients has not been described. The results suggest that patients with IH resolution have a good long-term prognosis. Most often IH resolution occurred while the dog was undergoing stabilization for heartworm extraction. When IHs are present, heartworm extraction should still be considered the treatment of choice and recommended as first-line therapy whenever possible.

## Data Availability

The data for this manuscript came from review of medical records and cannot be openly shared.

## Abbreviations

IH: Intracardiac heartworm
PO: By mouth
Q12: Every 12
Q8: Every eight
h: Hours
